# Supplementary material for: A bioactive peptide amidating enzyme is required for ciliogenesis
Source: eLife. 2017 May 17;6:e25728. doi: 10.7554/eLife.25728 (PMC5461114; doi:10.7554/eLife.25728)
Supplement: Supplementary file 1. — Transcript abundance of genes encoding intraflagellar transport (IFT), transition zone (TZ), Bardet-Biedl syndrome (BBS) and trafficking components in three control and three PAM amiRNA strains. Mean ± SD RPKM (reads per kilobase per million mapped reads) values from control and PAM amiRNA strains are tabulated. DOI: http://dx.doi.org/10.7554/eLife.25728.025 [file elife-25728-supp1.docx]

**Supplementary File 1. Changes in gene expression of ciliary components in control and PAM-amiRNA cells analyzed by RNA sequencing.**

|  |  |  | **EV control** | | **PAM KD** | |  |  |
| --- | --- | --- | --- | --- | --- | --- | --- | --- |
|  | **Protein name** | **transcript** | **Mean RPKM** | **Std dev** | **Mean RPKM** | **Std dev** | **Signifi-cantly different?** | **t-test** |
|  | PAM | Cre03.g152850.t1.2 | 21.24 | 1.18 | 6.05 | 0.14 | YES | 0.002 |
| IFT-A | IFT144 | Cre13.g572700.t1.2 | 13.68 | 1.90 | 11.90 | 1.63 | NO | 0.287 |
|  | IFT140 | Cre08.g362650.t1.1 | 8.27 | 1.08 | 8.01 | 1.18 | NO | 0.792 |
|  | IFT139 | Cre06.g268800.t1.2 | 12.19 | 2.24 | 10.88 | 0.86 | NO | 0.425 |
|  | IFT121 | Cre11.g475000.t1.1 | 11.83 | 1.23 | 11.04 | 1.50 | NO | 0.521 |
|  | IFT122 | Cre01.g065822.t1.1 | 14.90 | 1.92 | 13.10 | 1.97 | NO | 0.319 |
|  | IFT43 | Cre06.g251200.t1.2 | 10.82 | 1.74 | 9.42 | 1.38 | NO | 0.338 |
| IFT-B | IFT172 | Cre17.g703900.t1.2 | 10.70 | 2.90 | 7.73 | 1.44 | NO | 0.212 |
|  | IFT88 | Cre07.g335750.t1.1 | 13.14 | 1.87 | 11.49 | 1.50 | NO | 0.305 |
|  | IFT81 | Cre17.g723600.t1.2 | 10.29 | 3.38 | 7.89 | 1.24 | NO | 0.347 |
|  | IFT80 | Cre03.g204150.t1.2 | 13.53 | 3.49 | 10.84 | 1.27 | NO | 0.314 |
|  | IFT72/74 | Cre01.g027950.t1.2 | 16.43 | 3.63 | 13.04 | 1.28 | NO | 0.243 |
|  | IFT25 | Cre10.g450350.t1.2 | 12.46 | 3.50 | 10.41 | 1.30 | NO | 0.424 |
|  | IFT57 | Cre10.g467000.t1.2 | 14.56 | 3.53 | 13.57 | 1.34 | NO | 0.683 |
|  | IFT52 | Cre04.g219250.t1.2 | 18.58 | 4.20 | 15.32 | 1.84 | NO | 0.313 |
|  | IFT46 | Cre05.g241637.t1.1 | 22.88 | 6.22 | 20.57 | 1.99 | NO | 0.593 |
|  | IFT27 | Cre01.g047950.t1.1 | 12.79 | 2.53 | 14.21 | 1.91 | NO | 0.484 |
|  | IFT20 | Cre02.g089950.t1.2 | 18.30 | 4.60 | 19.02 | 1.35 | NO | 0.816 |
|  | IFT22 | Cre01.g039200.t1.2 | 14.35 | 2.08 | 12.94 | 2.07 | NO | 0.452 |
| TZ | CEP290 | Cre03.g167550.t1.1 | 3.41 | 1.08 | 4.21 | 0.59 | NO | 0.340 |
|  | NPHP4 | Cre12.g531400.t1.1 | 6.08 | 2.08 | 8.22 | 1.31 | NO | 0.219 |
| BBSome | BBS1 | Cre17.g741950.t1.1 | 10.83 | 0.34 | 10.65 | 0.34 | NO | 0.545 |
|  | BBS2 | Cre06.g257250.t1.1 | 9.18 | 0.37 | 8.32 | 0.11 | YES | 0.048 |
|  | BBS3 | Cre16.g664500.t1.2 | 7.30 | 1.68 | 7.99 | 0.47 | NO | 0.555 |
|  | BBS4 | Cre12.g548650.t1.1 | 4.77 | 1.16 | 4.31 | 0.16 | NO | 0.568 |
|  | BBS5 | Cre06.g267550.t1.2 | 7.11 | 0.16 | 7.95 | 0.61 | NO | 0.132 |
|  | BBS7 | Cre01.g043750.t1.1 | 7.96 | 0.56 | 7.94 | 0.27 | NO | 0.954 |
|  | BBS9 | Cre04.g219700.t1.1 | 5.29 | 0.13 | 5.90 | 0.27 | YES | 0.038 |
|  | BBS8 | Cre16.g666500.t1.2 | 10.28 | 0.22 | 10.77 | 0.35 | NO | 0.124 |
| Trafficking | Clathrin heavy chain | Cre02.g101400.t1.2 | 55.03 | 2.70 | 57.43 | 0.66 | NO | 0.261 |
|  | Clathrin light chain | Cre03.g155650.t1.2 | 58.62 | 5.96 | 60.74 | 3.66 | NO | 0.634 |
|  | Arf1 | Cre02.g142687.t1.1 | 439.62 | 27.14 | 450.96 | 13.84 | NO | 0.565 |
| Axonemal | RSP1 | Cre03.g201900.t1.1 | 7.67 | 2.17 | 5.10 | 0.93 | NO | 0.165 |
|  | RSP2 | Cre10.g427300.t1.2 | 6.01 | 1.91 | 3.80 | 0.39 | NO | 0.180 |
|  | TUA1 (apha tubulin 1) | Cre03.g190950.t1.2 | 709.17 | 76.87 | 754.24 | 85.97 | NO | 0.536 |
|  | TUA2 (alpha tubulin 2) | Cre04.g216850.t1.2 | 568.12 | 52.71 | 649.58 | 33.95 | NO | 0.099 |
|  | TUB1 (beta tubulin 1) | Cre12.g542250.t1.1 | 343.58 | 42.58 | 339.99 | 23.46 | NO | 0.906 |
|  | TUB2 (beta tubulin 2) | Cre12.g549550.t1.2 | 544.09 | 32.45 | 667.30 | 64.29 | NO | 0.060 |
|  | Gamma tubulin | Cre06.g299300.t1.2 | 14.88 | 2.80 | 19.72 | 0.64 | NO | 0.089 |
| Secreted | FEA1 | Cre12.g546550.t1.1 | 692.21 | 166.89 | 1239.12 | 260.08 | YES | 0.046 |
|  | FEA2 | Cre12.g546600.t1.1 | 5.59 | 1.52 | 9.90 | 7.08 | NO | 0.403 |
|  | Alkaline phosphatase | Cre08.g359300.t1.2 | 2.80 | 2.72 | 5.27 | 0.49 | NO | 0.254 |

Transcript abundance of genes encoding intraflagellar transport (IFT), transition zone (TZ), Bardet-Biedl syndrome (BBS) and trafficking components in three control and three PAM amiRNA strains. Mean ± SD RPKM (reads per kilobase per million mapped reads) values from control and PAM amiRNA strains are tabulated.
