# Supplementary material for: A bioactive peptide amidating enzyme is required for ciliogenesis
Source: eLife. 2017 May 17;6:e25728. doi: 10.7554/eLife.25728 (PMC5461114; doi:10.7554/eLife.25728)
Supplement: Supplementary file 2. — The source of each of the antibodies used in this study and the dilutions employed for immunofluorescence (IF) and western blot (WB) analysis are tabulated. DOI: http://dx.doi.org/10.7554/eLife.25728.026 [file elife-25728-supp2.docx]

| **Supplementary File 2.** **Antibodies used in this study.** | | |
| --- | --- | --- |
| **Antibody** | **Dilution** | **Source** |
| CrPAM CD | 1:500 (IF), 1:1000 (WB) | (1) |
| CrPAM lum | 1:500 (IF), 1:1000 (WB) | This study |
| α-tubulin | 1:1000 (WB, IF) | Thermo Fisher (B-5-1-2) |
| Poly-glutamylated tubulin | 1:500 (WB) | Adipogen (GT335) |
| Acetylated α-tubulin | 1:2000 (IF, WB) | Santa Cruz (611-B-1) |
| IFT81 | 1:500 (WB), 1:200 (IF) | Dr. Dennis Diener |
| IFT72/74 | 1:2500 (WB), 1:1000 (IF) | Dr. Dennis Diener |
| IFT46 | 1:1000 (WB), 1:500 (IF) | (2) |
| IFT139 | 1:500 (WB) | Dr. Douglas Cole |
| IC2 | 1:5000 (WB) | (3) |
| CEP290 | 1:500 (WB), 1:200 (IF) | (4) |
| NPHP4 | 1:200 (WB) | (5) |
| Clathrin | 1:1000 (WB) | Agrisera (AS10 690) |
| Arf 1 | 1:5000 (WB) | Agrisera (AS08 325) |

**REFERENCES**

1. Kumar D, Blaby‐Haas CE, Merchant SS, Mains RE, King SM, Eipper BA. Early eukaryotic origins for cilia-associatedbioactive peptide‐amidating activity. J Cell Sci 2016;129(5):943‐56.

2. Hou Y, Qin H, Follit JA, Pazour GJ, Rosenbaum JL, Witman GB. Functional analysis of an individual IFT

protein: IFT46 is required for transport of outer dynein arms into flagella. J Cell Biol. 2007;176(5):653‐65.

3. King SM, Otter T, Witman GB. Characterization of monoclonal antibodies against *Chlamydomonas*

flagellar dyneins by high‐resolution protein blotting. Proc Natl Acad Sci U S A. 1985;82(14):4717‐21.

4. Craige B, Tsao CC, Diener DR, Hou Y, Lechtreck KF, Rosenbaum JL, et al. CEP290 tethers flagellar

transition zone microtubules to the membrane and regulates flagellar protein content. J Cell Biol.

2010;190(5):927‐40.

5. Awata J, Takada S, Standley C, Lechtreck KF, Bellve KD, Pazour GJ, et al. NPHP4 controls ciliary trafficking

of membrane proteins and large soluble proteins at the transition zone. J Cell Sci. 2014;127(Pt 21):4714‐

27.
